# A Natural Course From Primary Intraocular Lymphoma to Brain Lymphoma in Four Years According to Patient's Choice

**DOI:** 10.7759/cureus.81476

**Published:** 2025-03-30

**Authors:** Toshihiko Matsuo, Takehiro Tanaka, Joji Ishida, Shotaro Kondo, Ken-ichi Matsuoka

**Affiliations:** 1 Department of Ophthalmology, Graduate School of Interdisciplinary Science and Engineering in Health Systems, Okayama University, Okayama, JPN; 2 Department of Ophthalmology, Okayama University Hospital, Okayama, JPN; 3 Department of Pathology, Graduate School of Medicine, Dentistry, and Pharmaceutical Sciences, Okayama University, Okayama, JPN; 4 Department of Neurological Surgery, Graduate School of Medicine, Dentistry, and Pharmaceutical Sciences, Okayama University, Okayama, JPN; 5 Department of Internal Medicine, Kurashiki Municipal Hospital, Kurashiki, JPN; 6 Department of Hematology and Oncology, Graduate School of Medicine, Dentistry, and Pharmaceutical Sciences, Okayama University, Okayama, JPN; 7 Department of Hematology, Endocrinology, and Metabolism, Institute of Biomedical Sciences, Tokushima University Graduate School, Tokushima, JPN

**Keywords:** brain biopsy, cell block pathology, diffuse large b-cell lymphoma, natural course, primary central nervous system lymphoma, primary intraocular (vitreoretinal) lymphoma, vitrectomy, vitreous opacity

## Abstract

Primary intraocular lymphoma or vitreoretinal lymphoma is a rare entity of diffuse large B-cell lymphoma that presents vitreous opacity and retinal and choroidal infiltration. Primary central nervous system lymphoma would occur previously, later, or concurrently with respect to primary intraocular lymphoma. This study reported a 72-year-old patient with a pathological diagnosis of primary intraocular lymphoma who developed central nervous system lymphoma four years later in the course of no treatment. She presented with a four-year history of blurred vision in both eyes after cataract surgeries. Three weeks previously, she underwent a vitrectomy in the left eye at a clinic, and measurements of the vitreous fluid showed a high level of interleukin-10 at 5739 pg/mL, in contrast with interleukin-6 at 142 pg/mL. Cytology of the vitreous fluid was class III on the Papanicolaou classification. Head magnetic resonance imaging detected nothing abnormal. She underwent vitrectomy in the right eye as a diagnostic procedure to show large cells in the vitreous which were positive for CD20 and Ki-67 and negative for CD3, leading to a pathological diagnosis of large B-cell lymphoma. Prophylactic chemotherapy with high-dose methotrexate was recommended as a therapeutic option, but she chose observation since she did not have any eye or systemic symptoms. In the follow-up every three months by an oncologist and an ophthalmologist, she did not have any symptoms, and serum levels of soluble interleukin-2 receptor were in the normal range at each visit. She was well for four years until the age of 76 years when she fell and hit her head, and an emergency head computed tomography scan showed a mass in the left occipital lobe. Magnetic resonance imaging demonstrated a well-defined circular mass in the left occipital lobe with a hyperintense signal in the T2-weighted fluid-attenuated inversion recovery (FLAIR) image and diffusion-weighted image. Fluorodeoxyglucose positron emission tomography showed no abnormal uptake systemically, except for the left occipital lesion. She underwent a brain biopsy by craniotomy to pathologically prove diffuse large B-cell lymphoma. She was recommended to receive first-line chemotherapy as the standard treatment but chose observation with no treatment and died of brain lymphoma nine months later. This case happened to illustrate a natural course of primary intraocular lymphoma which proceeded to central nervous system lymphoma four years later.

## Introduction

Lymphoma or malignant lymphoma is an uncontrolled proliferation of lymphocytes and classified largely into two systems, B-cell lymphoma and NK-/T-cell lymphoma, and further into subclasses based on the origin of lymphoma cells. Primary central nervous system lymphoma is a rare type of diffuse large B-cell lymphoma as a subclass of B-cell lymphoma with high malignancy which involves primarily the central nervous system and also the intraocular tissue [1-4]. The standard of care has been established to be high-dose methotrexate-based chemotherapy as induction therapy in a first-line therapy. In case of relapse, autologous peripheral blood hematopoietic stem cell transplantation after thiotepa-based high-dose chemotherapy for myeloablation is recommended in a second-line therapy or as consolidation therapy in case of untreated primary central nervous system lymphoma [5-7]. Oral administration of Bruton's tyrosine kinase inhibitors such as tirabrutinib to inhibit B-cell proliferation, whole-brain radiation, and chimera antigen receptor T-cell therapy to attack CD19-baring B cells is chosen in case of further relapse [8,9]. Most patients undergo the first-line chemotherapy which is known to be effective in inducing complete remission. In older patients, the dose was adjusted to reduce drug-induced toxicity. However, still a high rate of relapse after the first-line therapy is a major problem for patients and doctors.

Primary intraocular lymphoma, or vitreoretinal lymphoma in a recent term, is an even rarer entity of diffuse large B-cell lymphoma which presents vitreous opacity, retinal infiltration, or choroidal infiltration mainly in the subretinal pigment epithelial space or their combination [10-13]. The therapeutic strategy for intraocular lymphoma, which occurs concurrently with or in the course of primary central nervous system lymphoma, is the same as central nervous system lymphoma [14,15]. In contrast, the treatment strategy remains to be determined as for primary intraocular lymphoma which occurs at first and is not associated with central nervous system lymphoma for a while [14,16]. In the era of standard regimens of chemotherapy which have been established concretely, the natural courses of lymphoma in general have not been available as a matter of course. To get a hint for the management of a rare disease like primary intraocular lymphoma, this study documented a rare natural course of primary intraocular lymphoma which progressed to central nervous system lymphoma in the course of observation with no treatment in four years.

## Case presentation

A 72-year-old woman with a four-year history of blurred vision was referred to a university hospital due to suspicion of intraocular lymphoma. She had undergone cataract surgeries with intraocular lens implantation in both eyes five years previously at an eye clinic and had gained good visual acuity in both eyes. Three weeks previously, she underwent a vitrectomy in the left eye at the same clinic, and measurements of the vitreous fluid showed a high level of interleukin-10 at 5739 pg/mL, in contrast with interleukin-6 at 142 pg/mL. Cytology of the vitreous fluid was class III on the Papanicolaou classification. Head magnetic resonance imaging in another hospital detected nothing abnormal. She had been taking pemafibrate 0.2 mg daily and ezetimibe 10 mg daily for dyslipidemia, losartan 50 mg daily for hypertension, and febuxostat 10 mg for hyperuricemia for a decade. She had no other past history and did not smoke or drink alcohol. Physical examinations, including neurological examinations, showed nothing particular to be noted. Complete blood cell counts and blood chemistry tests were all in the normal range (Table 1). The urinalysis was also normal. A serum level of soluble interleukin-2 receptor was normal at 320.6 U/mL (normal range: 156.6-474.5).

**Table 1:** Blood examinations at the age of 72 years with intraocular lymphoma and at the age of 76 years with brain lymphoma Normal ranges at the in-house laboratory were for the time at 72 years. All values are reported in standard units. LD: lactate dehydrogenase; AST: aspartate aminotransferase; ALT: alanine aminotransferase; γ-GT: γ-glutamyl transferase; eGFR: estimated glomerular filtration rate; CRP: C-reactive protein; sIL-2R: soluble interleukin-2 receptor

	Normal range	At 72 years	At 76 years
Red blood cells (×10^6^/µL)	3.86-4.92	4.46	4.32
Platelets (×10^3^/µL)	158-348	311	304
White blood cells (×10^3^/µL)	3.30-8.60	6.23	4.45
Neutrophils (%)	40.0-70.0	58.3	61.5
Lymphocytes (%)	16.5-49.5	31.4	27.6
Monocytes (%)	2.0-10.0	4.8	8.3
Eosinophils (%)	0.0-8.5	4.9	2.0
Basophils (%)	0.0-2.5	0.6	0.6
Hemoglobin (g/dL)	11.6-14.8	12.8	12.4
Hematocrit (%)	35.1-44.4	39.7	39.4
Total protein (g/dL)	6.6-8.1	7.3	7.1
Albumin (g/dL)	4.1-5.1	4.3	3.9
LD (U/L)	124-222	174	172
AST (U/L)	13-30	15	25
ALT (U/L)	7-23	9	14
γ-GT (U/L)	9-32	10	11
Total bilirubin (mg/dL)	0.40-1.50	0.53	0.44
Urea nitrogen (mg/dL)	8.0-20.0	17.3	20.7
Creatinine (mg/dL)	0.46-0.79	0.63	0.60
eGFR (mL/min/1.73 m^2^)	60 or greater	69.6	72.3
Uric acid (mg/dL)	2.6-5.5	3.7	5.7
Total cholesterol (mg/dL)	142-248	208	190
Postprandial blood glucose (mg/dL)	<140	100	110
CRP (mg/dL)	0.00-0.14	0.12	0.86
Sodium	138-145	141	139
Potassium	3.6-4.8	4.0	4.1
Chloride	101-108	107	106
sIL-2R (U/mL)	156.6-474.5	320.6	467.2

**Figure 1:**
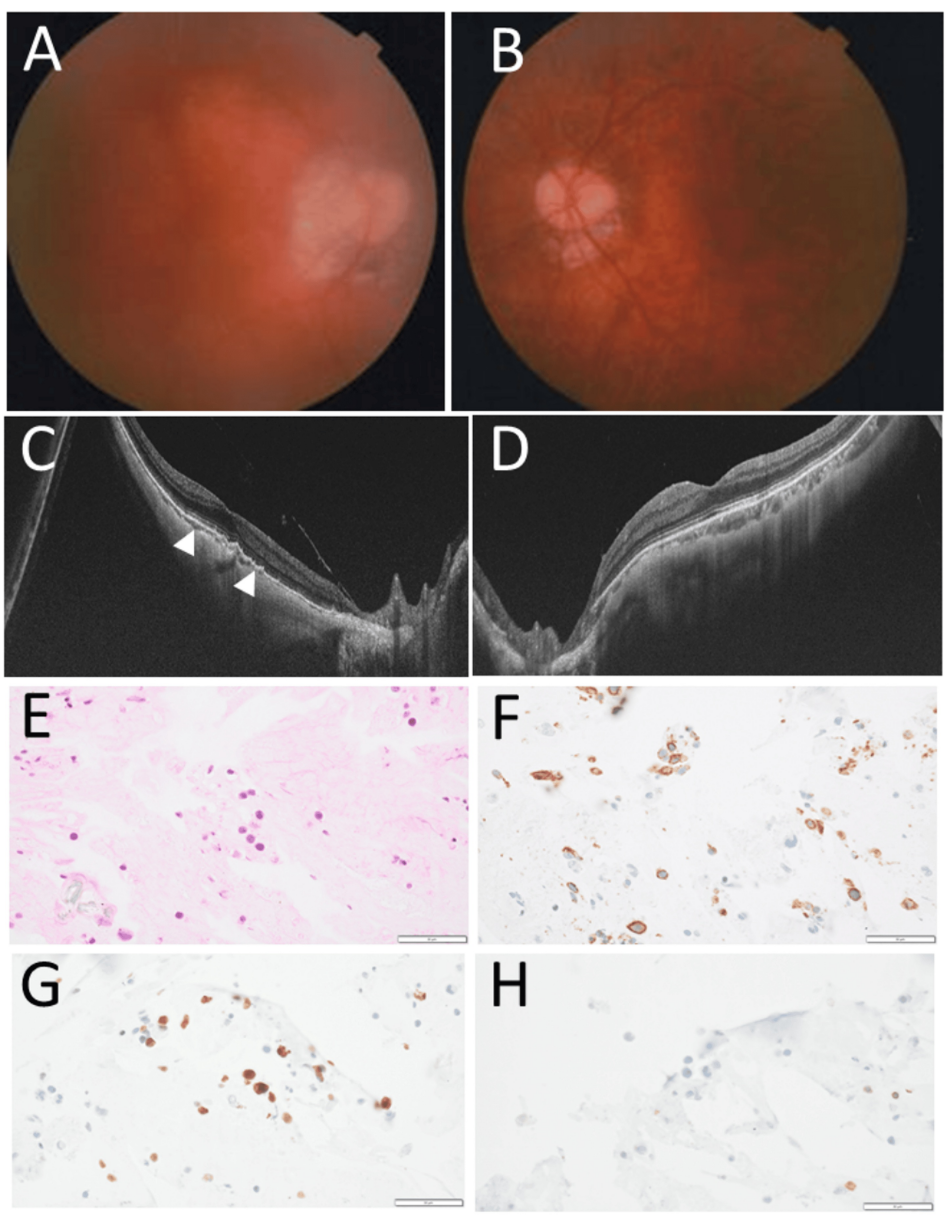
Fundus photographs, optical coherence tomography, and vitrectomy cell-block pathology at the age of 72 years Diffuse vitreous opacity in the right eye (A) and clear vitreous after vitrectomy in the left eye (B) at the age of 72 years. Horizontal sections of optical coherence tomography showing bumpy retinal pigment epithelial reflex line (arrowheads) with obscure photoreceptor ellipsoid zone due to high-myopic choroidal degeneration in the right eye (C), in contrast with relatively normal appearance in the left eye (D). Hematoxylin-eosin stain (A) and immunostains of cell-block sections of the entire vitreous fluid obtained by vitrectomy in the right eye. Large cells (E) are positive for CD20 (F) and Ki-67 (G) and negative for CD3 (H). Bar=50 µm.

At referral, the best-corrected visual acuity in decimals was 0.6 in the right eye and 1.2 in the left eye. The intraocular pressure was 17 mmHg in the right eye and 15 mmHg in the left eye. She had intraocular lens implantation in both eyes and showed diffuse mild vitreous opacity in the right eye (Figure 1A), in contrast with clear vitreous in the left eye (Figure 1B) after a vitrectomy that was done three weeks previously. The fundus in both eyes had no apparent active lesions except for myopic choroidal thinning around the optic disc to the macula (Figure 1C, 1D). She underwent a vitrectomy in the right eye as a diagnostic procedure. This time, the entire volume of the vitrectomy fluid was fixed lightly with the addition of one-tenth volume of 4% paraformaldehyde in phosphate-buffered saline and centrifuged to make a cell pellet [12,13]. The cell pellet was further fixed with 4% paraformaldehyde and embedded in paraffin to make a cell block to proceed to sectioning for immunostaining. Large cells (Figure 1E) in the vitreous were positive for CD20 (Figure 1F) and Ki-67 (Figure 1G) and negative for CD3 (Figure 1H) and were diagnosed as large B-cell lymphoma. The best-corrected visual acuity in the right eye was 0.7. Prophylactic chemotherapy with high-dose methotrexate was recommended as a therapeutic option, but she chose observation and also did not wish to have local therapy of intravitreal methotrexate injection since she did not have any eye or systemic symptoms.

**Figure 2:**
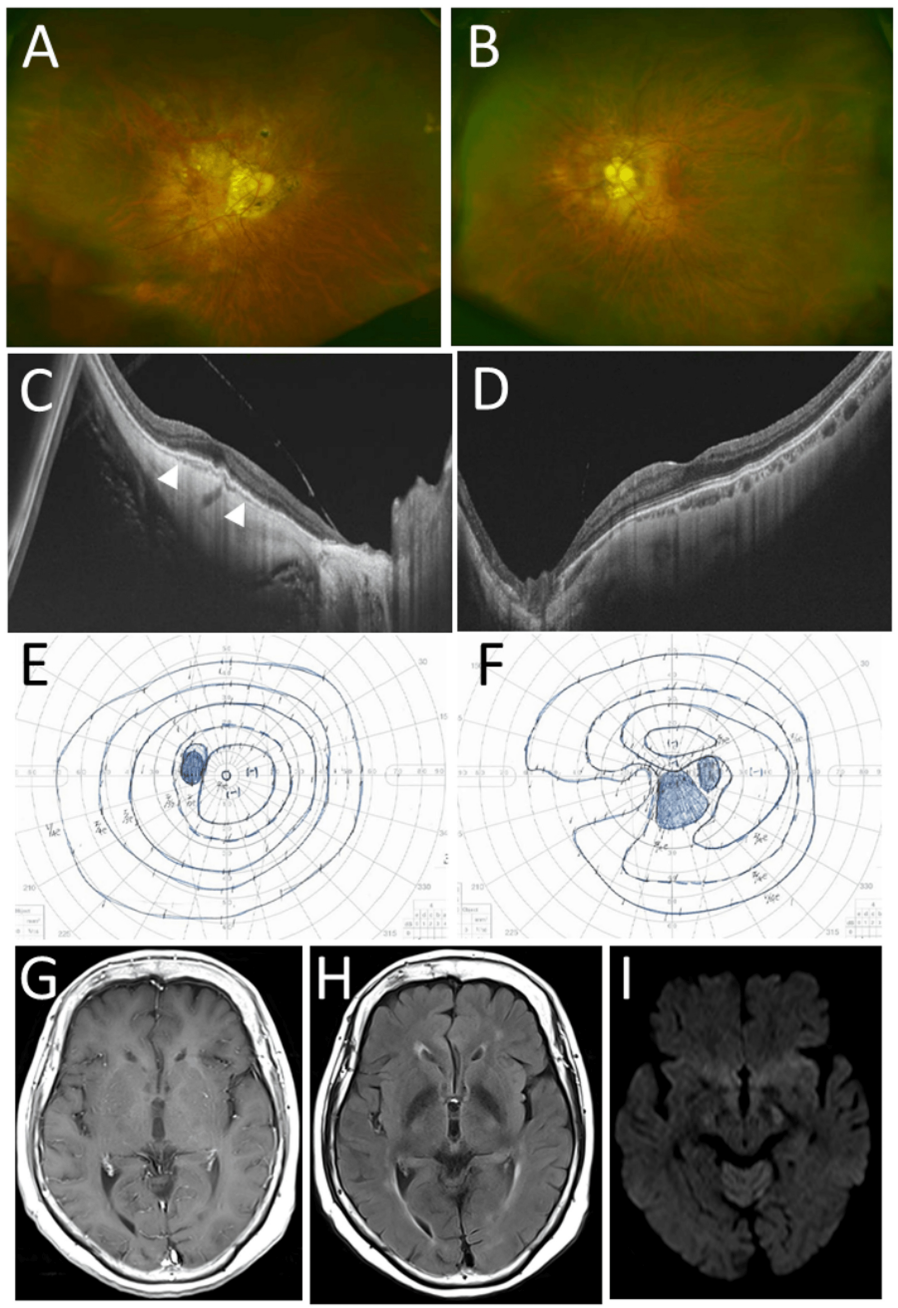
Fundus photographs, optical coherence tomography, Goldmann perimetry, and head magnetic resonance imaging at the age of 75 years Wide-field fundus photographs (A: right eye; B: left eye) and horizontal sections of optical coherence tomography (C: right eye; D: left eye) at the age of 75 years when she noticed a defect in the center of the right vision. Note high-myopic macular degeneration with the loss of the photoreceptor ellipsoid zone (arrowheads) in the right eye, the same as Figure 1C. Goldmann perimetry (E: left eye; F: right eye) showing central scotoma which deviates inferiorly in association with a glaucomatous nasal rupture-like defect (F) in the right eye. Magnetic resonance imaging (G: T1-weighted image with contrast enhancement; H: T2-weighted FLAIR image; I: diffusion-weighted image) showing no lesion, compared with the left occipital lesion a year later (Figure 3A, 3B). FLAIR: fluid-attenuated inversion recovery

She was followed every three months by an oncologist and an ophthalmologist. Two months later, she developed 3+ mutton-fat keratic precipitates in the left eye in contrast with no keratic precipitates in the right eye. The intraocular pressure was 20 mmHg in the right eye and 23 mmHg in the left eye. She began to have topical 0.1% betamethasone four times daily and 0.5% timolol twice daily in both eyes. The keratic precipitates in the left eye disappeared in a month, and afterwards, she continued 0.1% betamethasone twice daily and 0.5% timolol twice daily in both eyes. She did not have any symptoms, and serum levels of soluble interleukin-2 receptor were in the normal range at each visit. The anterior chamber and the vitreous in both eyes were clear with no new retinal or choroidal lesions. She was well for three years until the age of 75 years when she noticed a visual field defect on the nasal side in the right eye. The best-corrected visual acuity in the right eye dropped to 0.1, in comparison to 0.5 which was recorded at a visit three months previously. The visual acuity in the left eye remained 1.2. The intraocular pressure was 16 mmHg in the right eye and 13 mmHg in the left eye. She showed clear vitreous and myopic choroidal thinning in the posterior pole of the right eye (Figure 2A, 2C) which was the same as that at the preceding visit (Figure 1C). The posterior pole in the left eye appeared normal with milder myopic change, compared with the right eye (Figure 2B, 2D). Goldmann perimetry showed central scotoma which deviated inferiorly in association with a glaucomatous nasal rupture-like defect (Figure 2F). The visual field in the left eye was normal (Figure 2E). Head magnetic resonance imaging showed nothing abnormal (Figure 2G, 2H, 2I). The visual field defect in the right eye was attributed to myopic and glaucomatous changes, and she was given combination eye drops of 0.005% latanoprost and 0.5% timolol in place of 0.5% timolol in both eyes.

**Figure 3:**
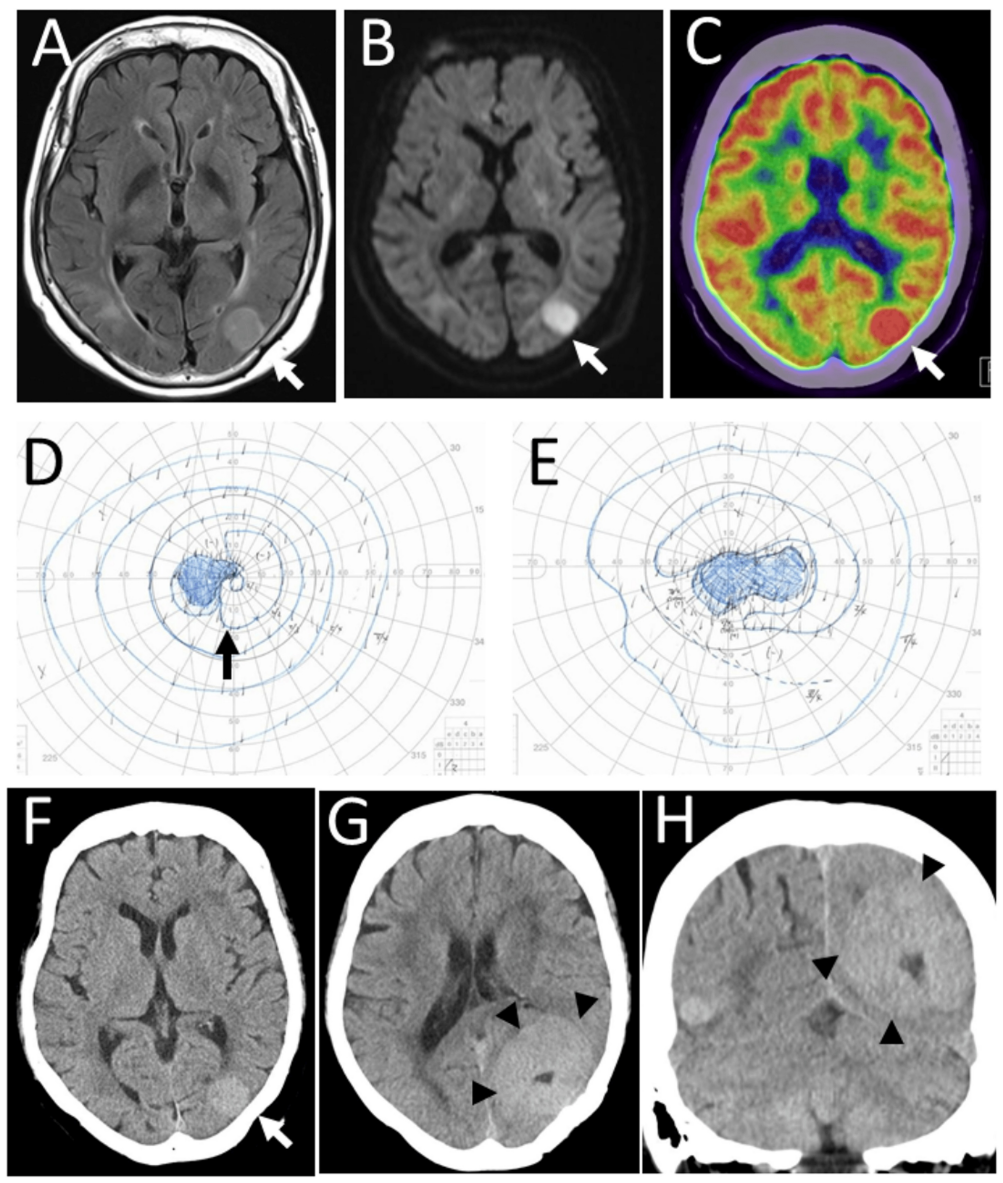
Magnetic resonance imaging, positron emission tomography, Goldmann perimetry, and computed tomography at the age of 76 years Magnetic resonance imaging at the age of 76 years showing a left occipital lesion (arrows) in T2-weighted FLAIR image (A) and diffusion-weighted image (B). Fluorodeoxyglucose positron emission tomography (C) showing high uptake (arrow) in the left occipital lesion. Goldmann perimetry (D: left eye; E: right eye) showing hemianopsia-like change (arrow) in the central vision of the left eye (D) and large central scotoma in the right eye (E). Computed tomography scans: the left occipital lesion (arrow) with no hemorrhage (F) one week after brain biopsy and the enlarged left occipital lesion (arrowheads) half a year later (G: axial image; H: coronal image). FLAIR: fluid-attenuated inversion recovery

**Figure 4:**
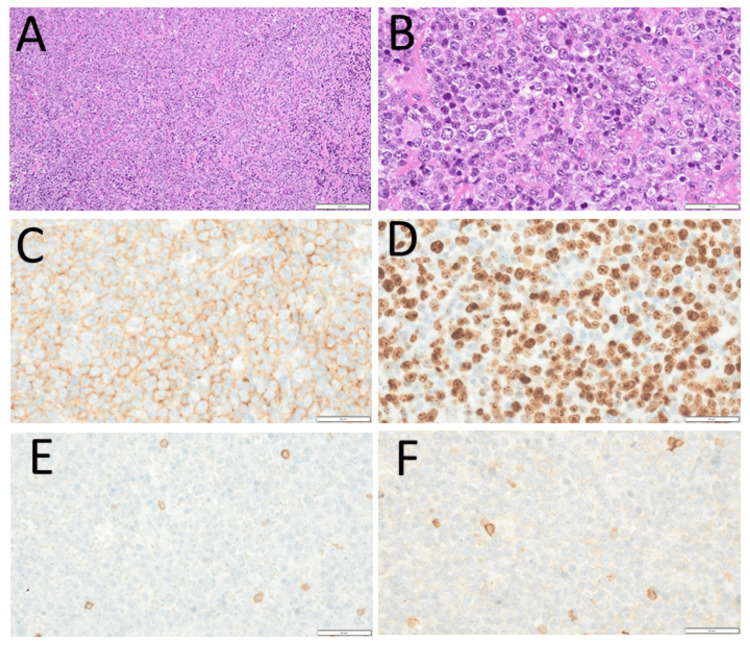
Pathology of brain biopsy at the age of 76 years Hematoxylin-eosin stains (A, B) and immunostains of brain biopsy specimen approached by craniotomy at the age of 76 years. Large anomalous cells in diffuse pattern of infiltration are positive for CD20 (C) and Ki-67 (D) and negative for CD3 (E). There are only a few CD5-positive large cells (F). Bar=50 µm, except for A which is 200 µm.

One year later, at the age of 76 years, she fell and hit her head. She did not lose consciousness and did not have any neurological symptoms. An emergency head computed tomography scan showed a mass in the left occipital lobe. Magnetic resonance imaging demonstrated a well-defined circular mass in the left occipital lobe with a hyperintense signal in the T2-weighted fluid-attenuated inversion recovery (FLAIR) image (Figure 3A) and diffusion-weighted image (Figure 3B). Fluorodeoxyglucose positron emission tomography showed no abnormal uptake systemically, except for the left occipital lesion (Figure 3C). A serum level of soluble interleukin-2 receptor was normal at 467.2 U/mL (Table 1). The best-corrected visual acuity was 0.1 in the right eye and 1.0 in the left eye, the same as that one year previously. Goldmann perimetry showed a hemianopsia-like change in the central vision of the left eye (Figure 3D) and large central scotoma in the right eye (Figure 3E). She underwent a brain biopsy by craniotomy with no complications (Figure 3F) to pathologically prove diffuse large B-cell lymphoma (Figure 4A-4F). She was recommended to receive first-line chemotherapy as the standard treatment. She, however, chose the observation with no treatment after several rounds of dialogue on different occasions with herself, her family, and medical professionals. Her main point of concern was that she would have a relatively high risk for relapse after complete remission which could be obtained by the first-line chemotherapy.

She was referred to a regional hospital for best supportive care at home with her family. In half a year, she maintained the visual acuity of 0.1 in the right eye and 0.8 in the left eye and continued topical 0.1% betamethasone and 0.5% timolol eye drops twice daily in both eyes. She had dizziness due mainly to right homonymous hemianopsia, general fatigue, and occasional nausea in the background of the enlarging lymphoma lesion in the left occipital lobe (Figure 3G, 3H). Occasional nausea was relieved by the temporary use of oral dexamethasone 4 mg daily, and night anxiety with insomnia was successfully coped with the daily use of oral alprazolam 0.2 mg. The best-corrected visual acuity was 0.01 in the right eye and 0.4 in the left eye eight months later from the diagnosis of brain lymphoma. She died nine months later from the diagnosis of brain lymphoma.

## Discussion

Primary intraocular or vitreoretinal lymphoma presents two points of difficulties in the clinical management: one is the diagnosis and the other is the treatment strategy. The pathological diagnosis of cancer, including lymphoma, remains the gold standard, and immunohistochemical staining is a key for establishing the classification of lymphoma. After the establishment of diagnosis, a therapeutic regimen is determined based on the pathological classification of lymphoma. The primary intraocular lymphoma manifests as vitreous opacity or subretinal pigment epithelial choroidal infiltration with retinal infiltration or their combination. In the field of ophthalmic practice, it is considered a low risk to obtain aqueous humor by anterior chamber puncture and to submit the sample for cytological examinations [16,17]. It is also a standard procedure to obtain the vitreous fluid for cytological examinations, measurement of interleukin-10 and interleukin-6, and flow cytometry. In this context, immunostaining of sections of a paraffin-embedded cell block, which is prepared by the spinning down of the vitrectomy fluid, is desirable to achieve more concrete evidence for the pathological diagnosis of lymphoma [8,12,18,19]. In the case of only choroidal infiltration with no vitreous opacity, choroidal biopsy would provide the pathological diagnosis of lymphoma even though the choroidal biopsy may have a risk for retinal detachment [20].

In case central nervous system lymphoma is detected after intraocular lymphoma has been diagnosed pathologically by cell-block sections of the vitrectomy fluid, treatment will follow the strategy for primary central nervous system lymphoma. On the other way around, if intraocular lymphoma is detected in the course of central nervous system lymphoma, systemic chemotherapy or local therapy such as whole-eye radiation will be considered, based on the treatment history for central nervous system lymphoma. It remains still questionable for patients with primary intraocular lymphoma to undergo prophylactic systemic chemotherapy to prevent the development of central nervous system lymphoma. Central nervous system lymphoma did develop in a patient with primary intraocular lymphoma who chose to undergo prophylactic systemic chemotherapy with high-dose methotrexate and cytarabine [8,13]. As for local therapies toward the eyes with intraocular lymphoma, whole-eye radiation is definitely effective to induce complete remission, while repeated intravitreal injections of methotrexate remain questionable to obtain local control and to prevent the future development of central nervous system lymphoma [12,15].

The present patient chose no prophylactic systemic chemotherapy and maintained no relapse in the eyes and no development of central nervous system lymphoma for four years. To our surprise, she did not choose to undergo the first-line chemotherapy when she developed central nervous system lymphoma. As expected, she died of brain lymphoma nine months later. She had lost her husband because of pneumonia a year previously and had a good understanding of her situation after the diagnosis of brain lymphoma. Her family also respected her decision and gave her every support to live at home until her death. In retrospect, it would be understandable for her to choose the natural course since the treatment does not have a guarantee to cure the disease. According to her choice, we could know a natural course of primary intraocular lymphoma to central nervous system lymphoma. The age of 76 years in the development of brain lymphoma would be one major factor for different interpretations. Since she was healthy and had no comorbidities, we were surprised at first to hear her decision not to have chemotherapy. In this sense, this study naturally has a limitation, like single-case bias.

According to our previous study of 22 consecutive patients with primary intraocular lymphoma diagnosed by immunostaining of vitrectomy cell blocks [13], 17 patients developed central nervous system lymphoma: three patients developed intraocular and central nervous system lymphoma simultaneously, nine patients developed central nervous system lymphoma one month to five years (median: three months) after intraocular lymphoma, and five patients developed central nervous system lymphoma preceding the diagnosis of intraocular lymphoma by three months to nine years and eight months (median: 1.5 years). In contrast, five patients did not develop central nervous system lymphoma: two patients did not develop local recurrence or central nervous system lymphoma in the follow-up period of five years and 11 years, respectively, after vitrectomy alone without additional local or systemic treatment. The remaining three patients with intraocular lymphoma had insufficient follow-up periods to determine the prognosis. The four-year interval in the present patient between the development of intraocular lymphoma and brain lymphoma is within the range as shown in the previous study [13]. Overall, patients with primary intraocular lymphoma, even though they have not yet developed central nervous system lymphoma in several months, should be followed regularly in the long term since they would have a risk for developing central nervous system lymphoma many years later.

## Conclusions

Primary intraocular lymphoma or vitreoretinal lymphoma presents two clinical questions that remain to be solved. The first one is the diagnosis, and the second is the treatment strategy. In contrast, primary central nervous system lymphoma has the standard procedure for the diagnosis by brain biopsy and the standard flow of treatment from the first-line and second-line therapy to the third-line therapy. The present 72-year-old woman who developed vitreous opacity with no retinal or choroidal manifestations in healthy condition was diagnosed pathologically as having primary intraocular lymphoma by immunostaining of cell-block sections prepared from the entire vitrectomy fluid. She did not choose prophylactic chemotherapy and developed brain lymphoma four years later. According to her wish, she did not receive standard chemotherapy for brain lymphoma and died nine months later. In retrospect, she showed a natural course of primary intraocular lymphoma to central nervous system lymphoma, and thus, the clinical course would give a hint for the diagnosis and standard treatment for primary intraocular lymphoma.
